# A prospective cohort study to assess seroprevalence, incidence, knowledge, attitudes and practices, willingness to pay for vaccine and related risk factors in dengue in a high incidence setting

**DOI:** 10.1186/s12879-016-2055-4

**Published:** 2016-11-25

**Authors:** Ruth Aralí Martínez-Vega, Alfonso J. Rodriguez-Morales, Yalil Tomás Bracho-Churio, Mirley Enith Castro-Salas, Fredy Galvis-Ovallos, Ronald Giovanny Díaz-Quijano, María Lucrecia Luna-González, Jaime E. Castellanos, José Ramos-Castañeda, Fredi Alexander Diaz-Quijano

**Affiliations:** 1 Organización Latinoamericana para el Fomento de la Investigación en Salud, Bucaramanga, Santander Colombia; 2 School of Medicine, Universidad de Santander, Bucaramanga, Santander Colombia; 3 Public Health and Infection Research Group, Faculty of Health Sciences, Universidad Tecnológica de Pereira, Pereira, Risaralda Colombia; 4 Grupo de Virología, Universidad El Bosque, Bogotá, Colombia; 5 Centro de Investigaciones sobre Enfermedades Infecciosas, Instituto Nacional de Salud Pública, Cuernavaca, Morelos, Mexico; 6 Department of Epidemiology, Universidade de São Paulo, São Paulo, São Paulo Brazil

**Keywords:** Dengue, Seroprevalence, Incidence, Knowledge, Vaccines, Risk factors, Colombia

## Abstract

**Background:**

Dengue is one of the most important vector-borne diseases in the world, causing significant morbidity and economic impact. In Colombia, dengue is a major public health problem. Departments of La Guajira, Cesar and Magdalena are dengue endemic areas. The objective of this research is to determine the seroprevalence and the incidence of dengue virus infection in the participating municipalities from these Departments, and also establish the association between individual and housing factors and vector indices with seroprevalence and incidence. We will also assess knowledge, attitudes and practices, and willingness-to-pay for dengue vaccine.

**Methods:**

A cohort study will be assembled with a clustered multistage sampling in 11 endemic municipalities. Approximately 1000 homes will be visited to enroll people older than one year who living in these areas, who will be followed for 1 year. Dengue virus infections will be evaluated using IgG indirect ELISA and IgM and IgG capture ELISA. Additionally, vector indices will be measured, and adult mosquitoes will be captured with aspirators. Ovitraps will be used for continuous estimation of vector density.

**Discussion:**

This research will generate necessary knowledge to design and implement strategies with a multidimensional approach that reduce dengue morbidity and mortality in La Guajira and other departments from Colombian Caribbean.

## Background

Although arboviral diseases, such as Chikungunya and Zika [1, 2], have recently emerged in the Americas, dengue is globally the most important of the group of three viruses, particularly related to both incidence and the burden of disease. An estimated average of 9221 dengue deaths occurred per year between 1990 and 2013, increasing from a nadir of 8277 (95% uncertainty estimate 5353–10,649) in 1992, to a peak of 11,302 (6790-13,722) in 2010 [3]. This yielded a total of 576,900 (330,000–701,200) years of life lost to premature mortality attributable to dengue in 2013 [3]. The incidence of dengue increased considerably between 1990 and 2013, with the number of cases more than doubling every decade, from 8.3 million (3.3 million–17.2 million) apparent cases in 1990, to 58.4 million (23.6 million–121.9 million) apparent cases in 2013 [3]. When disability from moderate and severe acute dengue, and post-dengue chronic fatigue are taken into account, 566,000 (186,000–1.415,000) years lived with disability were attributable to dengue in 2013 [3]. Combining fatal and non-fatal outcomes, dengue was responsible for 1.14 million (0.73 million–1.98 million) disability-adjusted life-years in 2013.

In the South American setting, the consequent costs of dengue are high. Dengue imposes a substantial economic and disease burden in Latin American countries, such as Brazil, Mexico and Colombia, among others [4–8]. In Brazil, the estimated cost for dengue to communities for the epidemic season of 2012–2013 was US$ 468 million (90% CL: 349–590) or US$ 1212 million (90% CL: 904–1526) after adjusting for under-reporting [6]. For Mexico, the annual cost, including surveillance and vector control, was US$170 (95% CL: 151–292) million, or $1.56 (95% CL: 1.38–2.68) per capita, comparable to other countries in the region, during 2010–2011. In that annual total, $87 (95% CL: 87–209) million or $0.80 per capita (95% CL: 0.62–1.12) is related to illness [8]. In Colombia, during the epidemic year 2010, 1198.73 DALYs were lost per million inhabitants versus 83.88 in endemic years were estimated [4]. The total financial cost of the disease to communities in Colombia was US$167.8 million for 2010, US$129.9 million for 2011, and US$131.7 million for 2012. The cost of mosquito prevention borne by households was a major cost driver, accounting for 46% of the overall cost in 2010, 62% in 2011, and 64% in 2012 [4].

In Colombia, dengue is endemic in most of the country (which is <2200 m.a.s.l.), consistent with the wide distribution of its main vector *Aedes aegypti* [9–12]. All the administrative levels report either autochthonous cases (>90% of the territories) or imported cases (as occurs in Bogota, the capital city). Between 2000 and 2011, the annual number of non-severe dengue disease cases reported in nationwide surveillance data ranged between 22,775 (2000) and 147,670 (2010) [13]. Across the period 2000–2011, the annual number of severe dengue disease cases reached a maximum of 9777 (38.3 per 100,000 population) in 2010, and a minimum of 1383 in 2011 [13]. In 2014, Colombia reporting 105,356 cases diagnosed by syndromic surveillance, of which 46,842 cases were confirmed by laboratory diagnosis, with 2619 severe cases and 166 deaths [14]. In 2015, there were 94,916 cases diagnosed by syndromic surveillance with 1360 severe cases and 72 deaths [15].

**Fig. 1:**
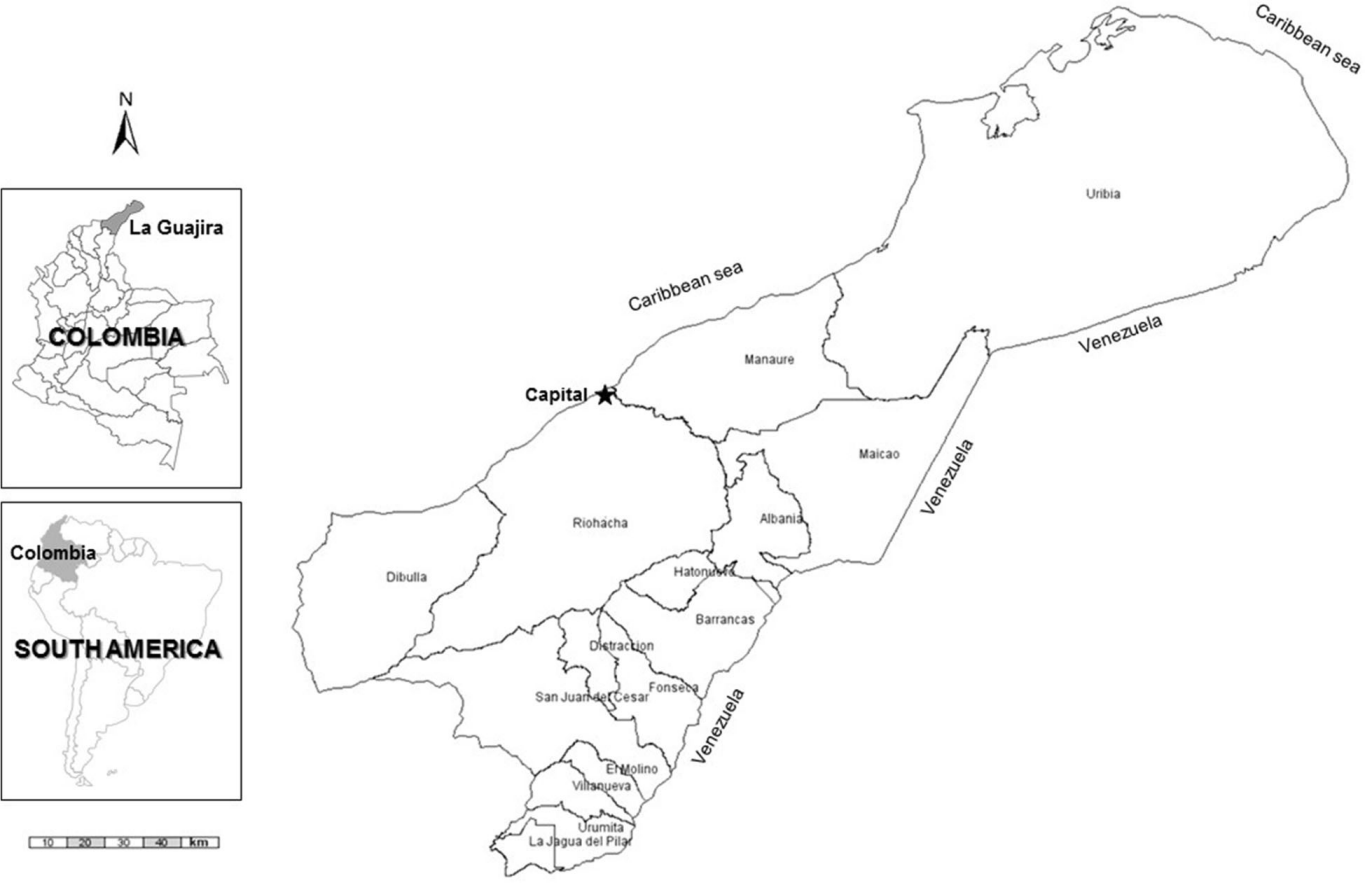
Relative position and map of La Guajira and its municipalities, Colombia, South America

**Fig. 2:**
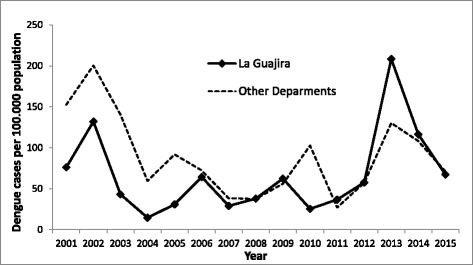
Dengue incidence rates in La Guajira compared with the other Departments from Colombia, 2001–2015

In Colombia, the Caribbean coastal region is particularly endemic for dengue. In this region the *La Guajira* department (Fig. 1), is located in the northeast, bordering Venezuela. This department reported more than 5000 cases between 2007 and 2013 (2033 in 2013), of which 250 were severe. Thus, the increase in dengue cases has been particularly evident in La Guajira, where incidence went from being below the national average to overcome the incidence observed in the other departments of Colombia (Fig. 2).

La Guajira is composed of 15 municipalities, all of which reported dengue cases during 2007–2013 (ranging 7–606 cases per year). In La Guajira, Riohacha, the, coastal capital city and Maicao (Fig. 1) reported more than 1500 and 1300 cases during this period, respectively.. In both cities an increasing trend was noted from 85 and 111 cases in 2007, to 591 and 606 cases in 2013. The La Guajira Department has had all the serotypes of Dengue virus (DENV) circulating since the 1980s [16].

Surveillance may be incomplete in this area of the country, and it is likely that dengue cases are under-recorded [16–18]. Enhanced active surveillance will improve the assessment of the real morbidity of dengue in La Guajira. There is a paucity of published epidemiological data with no relevant scientific publications on any aspect of dengue [19]. No research has been conducted to assess the various factors which drive dengue transmission in this setting [20]. Those factors include education, economic income [21], migration and population flows [22], cross-protection between serotypes [12, 23], and the environmental factors which have influenced dengue epidemiology, including climate change [24, 25], and housing standards [26–28]. All of these are important in order to develop effective integrated interventions oriented to reduce transmission and the burden of disease in a given area. Asymptomatic infections, are not usually assessed by surveillance systems, but significantly affect transmission [29]. Some studies have suggested that up to 80% of infected subjects show no signs of disease [29–31]. If all these factors are taken into account, interventions would likely to be more cost-effective.

The main objective of this research is to determine the seroprevalence and the incidence (outcome variables) of DENV infection in eleven municipalities (nine from La Guajira, one from Magdalena department and one from Cesar department), as well the association between them with individual, housing factors and vector indexes (independent variables). In addition, knowledge, attitudes and practices, as well as willingness to pay for a dengue vaccine, will also be assessed.

## Methods/design

### Design and population

A prospective cohort study will be nested with a cross-sectional study from a clustered multistage sampling in 11 endemic municipalities: nine in La Guajira (Riohacha, Albania, Fonseca, San Juan del Cesar, Distracción, Maicao, Villanueva, Uribia and Manaure), one of Magdalena (Retén) one of Cesar (Valledupar) to assess seroprevalence. Approximately 1000 homes will be visited to around 5000 people older than one year-old living in these areas will be invited to participate. Persons planning to change their place of residence in the next 6 months will be excluded.

Subjects will be followed during one year. DENV infection will be evaluated using serologic tests and, vector indexes will be measured. Adult mosquitoes will be captured with aspirators. Ovitraps will be used for continuous estimation of vector density.

### Sampling and sample size

A multistage cluster sampling will be performed. Initially there will be a random selection of blocks in each municipality. These will be visited to conduct a population census of houses. A random selection of households will be visited to invite all inhabitants to participate. When a family does not consent to participate, another house in the same block will be randomly selected. It is expected at least 1000 houses in the 11 participating municipalities will be included, which could represent a potential of up to 5000 individuals at an average of five persons per household.

With this sample size we expect to estimate a seroprevalence of 50% (proportion with the largest margin of error), with an error of 1.38%, with a design effect (DEFF) equal to 1, and of 1.95%, with a DEFF of 2 [32]. The whole projected population of the participating municipalities was considered as the universe in these calculations. The software used in sample size calculation was EPIDAT 3.1 (free).

## Variables

### Dependent: dengue incidence and seroprevalence

#### Incidence

Dengue incidence will be assessed by comparing the baseline sample (Visit 1) with follow-up (visits 2, 3 and 4, which are separated by 4 months) using specific tests for DENV, Panbio®, IgM capture ELISA (sensitivity 93.7% [95% CI 90–96] and specificity 87.8% [95% CI 82–93]) and IgG capture ELISA (sensitivity 72.8 [95% CI 67–78] and specificity 95.3% [95% CI 91–98]) [33]. IgG capture ELISA is calibrated in such way that a positive result corresponds with a HAI titer higher than 1:1280 which is considered suggestive of recent infection [34]. An incidence case will be defined as those negative at both tests in the baseline assessment and positive later by any test.

#### Seroprevalence

DENV seroprevalence will be determined in the baseline sample with the indirect ELISA test for IgG with the Panbio E-DEN 01G and IgM capture tests [35]. Panbio E-DEN 01G exhibited sensitivity of 100% and specificity of 98% for IgG detection in serum samples collected from patients with suspected acute DENV infections, living in dengue endemic areas, compared with serum samples from patients with other viral infections [33]. Moreover, this indirect IgG ELISA has been widely used in different studies from endemic and non-endemic areas to define seroprevalence status [36–40].

This will be performed following the manufacturer’s instructions. If any of the two tests is positive, the subject will be considered to have been previously infected with DENV.

As a measure of quality control, 100 serum samples will be randomly selected to validate the IgG test using PRNT as gold standard.

#### Independent variables

Individual characteristics such as age, sex, level of education, occupation and autorefered human mobility will be considered as independent variables. Housing characteristics, the availability of drinking water, the presence of nets at windows and doors, air conditioning, the number of individuals who live in the house and family structure, will also be assessed.

The relative abundance of the vector in homes will be assessed through the index of positive containers (# of infested containers/# of inspected water containers), the proportion of geographic areas calculating the Breteau index (# of positive containers/# of homes inspected), a house index (% of house with larvae or pupae) and ovitrap index. In addition, the extent of vector control performed by household members or by the secretaries of health of the municipalities (e.g. insecticide spraying) will be also measured.

Other variables, including clinical classification of symptomatic or asymptomatic infection, will be based on information provided weekly during active surveillance of fever cases in homes and performed by staff who will monitor the ovitrap will be also evaluated. These technicians will ask about any fever case in the family. Febrile patients will be interviewed by a nurse using a standardized questionnaire and taking a blood sample for diagnostic tests. Samples obtained from febrile patients will be test with CDC DENV-1-4 real-time RT-PCR assay and/or IgM ELISA (Panbio) depending of the day of blood collection.

Knowledge, attitudes and practices (KAP) on dengue will be evaluated using a questionnaire previously validated by Cáceres-Manrique et al. [41]. A questionnaire to assess willingness to pay for dengue vaccines was developed using previously published information from Vietnam, Thailand and Colombia [42]. A summary of the variables and the moment of evaluation are presented in Table 1.

**Table 1:** Variables and moments of evaluation

Information to be collected	Moment of evaluation
Demographic and mobility variables and history of symptoms in the last months	Baseline and every 4 months (visits 1, 2, 3 and 4).
Blood sampling for serological tests.	Baseline and every 4 months (visits 1, 2, 3 and 4).
Housing characteristics including number of cohabitants	Baseline
Infestation indexes	Baseline and every 4 months (visits 1, 2, 3 and 4).
Revision of ovitraps	Weekly, during a year since the baseline
Active febrile surveillance including blood sampling in febrile patients.	Weekly, during a year since the baseline
Knowledge, attitudes and practices (KAP).	Baseline
Questionnaire about willingness to pay for vaccine.	Baseline

#### Assessment and follow-up

**Fig. 3:**
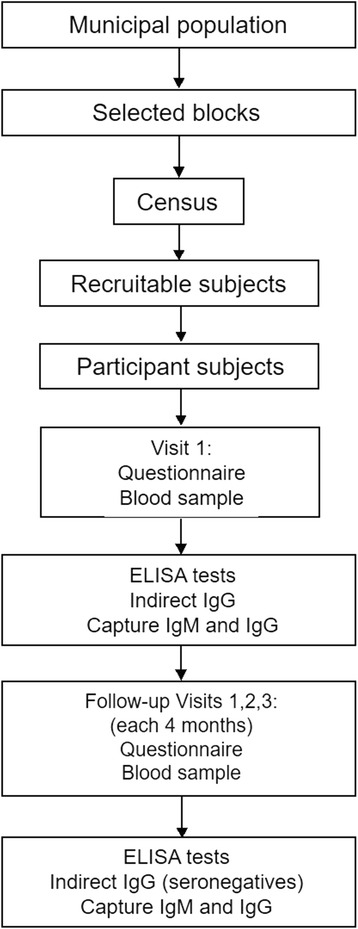
Assessment and follow-up of the cohort

Participating subjects will be evaluated and followed for 12 months (Fig. 3). At baseline (Visit 1) a structured interview will be conducted and a sample of blood (5–8.5 ml) will be taken. The questionnaire will include socio-demographic and mobility variables. A history of fever in the last 3 months and symptoms of dengue in the last 15 days will be recorded.

Three follow-up visits will be performed, one every 4 months over the year of the study. The follow-up structured questionnaire will include sociodemographic and mobility variables, but also gather information regarding attendance at healthcare services during the follow-up period. A blood sample to measure DENV antibodies will be taken at each of the three follow up visits.

Questions about history of Zika and/or Chikungunya, previous Yellow Fever vaccination and previous recent travel to other localities, were also included in the baseline and/or follow-up questionnaires.

Besides active surveillance of febrile cases, participants will be asked to attend the local allocated health center in the municipality for clinical evaluation if fever develops. Where dengue is suspected, a blood sample for a full blood count and dengue diagnosis, as previously detailed, will be drawn. This follow-up exercise will be carried out in coordination and collaboration with the Secretaries of Health of La Guajira and of the municipalities. Surveillance for identifying symptomatic cases will be enhanced.

#### Estimation of the vector relative abundance and housing factors assessment

On the first visit a structured survey on housing characteristics, family composition, and anti-vector measures will be performed. An inventory and inspection of the different types of water containers inside the house, in the yard and garden will be undertaken to determine whether they are infested with larvae or pupae. Breteau and household indices will be calculated utilizing information on the rates of positive containers. Each home will be georeferenced using a Garmin equipment (GPSMAP 64 s).

Calculations of the *Aedes* indexes will be made at each follow-up event (Visit 2, 3 and 4). Subjects will be asked about the anti-vector measures taken against the vector and inspection will be repeated to estimate indices.

Besides infestation indices based on larvae/pupae identification, a weekly surveillance of vector eggs will be conducted based in follow-up of ovitraps. Ovitraps will be placed in participant houses, developed with black wide-mouthed containers of 1 liter capacity. After testing in the Riohacha municipality (Fig. 1), and taking account of the high temperature and humidity in the area, it was decided that a qualitative filter paper strip Boeco® 10 cm wide and 800 ml of water will be placed in each ovitraps (unpublished data). The ovitrap will be reviewed weekly to collect neckband filter paper and replace filter paper. These strips will be transferred to the laboratory where the eggs will be then counted.

In addition, to assist with monitoring vector infection, adult mosquitoes will be collected using electric aspirators prokopack John W. Hock [43]. Collections will be made inside homes in rooms, living rooms, and bathrooms and around the home in the yard and garden. In each environment two cycles of aspiration of 5 min each will be made. At least an adult mosquito collection will be performed in every home. Specimens captured will be taken to the laboratory where speciation and sex separation will be done, using taxonomic keys.

#### Monitoring of circulating virus in the vector

**Fig. 4:**
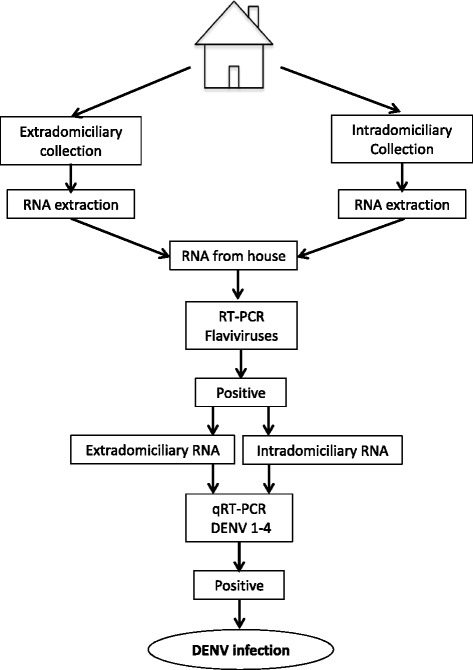
Diagnostic algorithm for dengue infection in mosquitoes

Female groups of *Aedes aegypti*, captured during visits to participating households, will be assessed in order to establish DENV infection, using a nested generic RT-PCR for flavivirus screening. [44]. Those recorded as positive will be evaluated with a real-time RT-PCR for DENV-1-4 (Fig. 4).

#### KAP on dengue and the willingness-to-pay for vaccines

On the first visit an adult in each homes, will be asked to answer the KAP survey to assess knowledge about the disease, attitude to illness compatible with dengue, as well as the activities needed to prevent disease. The structured surveys will also include willingness to pay for vaccines. For this purpose, the questionnaire was designed assuming two scenarios, one of 70% efficacy at 10 years and another 95% efficacy at 30 years, with a range of vaccination prices between COP$3000 and COP$900,000 (around US$1 and US$300, respectively), and two vaccination regimens, one of three doses and another utilizing a single dose. The evaluated prices are similar to those considered by other researchers in a previous study [42]. This questionnaire will include data on the mean family income.

#### Data collection and statistical analysis

To assist with data management, the forms for data collection for baseline and follow-up visits were designed. These forms were developed to determine the KAP and the willingness-to-pay for vaccines and for baseline and follow-up of subjects. These will be pilot tested, updated and used in the recruitment stage. An access database for the electronic storage of information for the project will be designed. Double digitation will be made of each available data collection form, and these will be cross-checked using the Data Compare Package of Epi Info (TM) 3.5.1. Differences for each field filled will be established, and paper printed to check data and arrive at an audited data base which will be the source for further analysis. Throughout the process of data management, periodic comparison of digital records and the final audited data bases will be made.

A description of the population, the characteristics of the houses, breeding sites and indexes will be made. Categorical variables will be recorded as frequencies. Measures of central tendency (mean or median as distribution) will be used for continuous variables. The seroprevalence will be determined and the estimate of the variance will consider the design effect and 95% confidence intervals obtained. The cumulative incidence and incidence density for both the total population and by age groups will be calculated using year age groups. The frequency of asymptomatic and symptomatic infections will be determined.

Bivariate analysis will be undertaken for each of the dependent variables and compared with possible independent variables. The Chi square test for categorical variables and the Student *t* test will be used for continuous variables with normal distribution or the Mann-Whitney test for continuous variables with *p* <0.05 in the normality test.

Those variables in the bivariate analysis achieving *p* < 0.20 will be evaluated with multivariate analysis binomial regression or multilevel analysis as appropriate. The modeling of the variables will be considering obtaining a parsimonious final model, including those variables that have statistical significance (*p* <0.05) or modifying the estimated primary endpoint by more than 10%. Analyses will be performed using Stata version 12.0 statistical program.

## Discussion

Implementing strategies to reduce morbidity and mortality from dengue in endemic areas requires integrated epidemiological information that would allow an improvement in the knowledge on factors associated with local transmission [45]. This project has a multidimensional approach, which includes human factors, the vector, the environment and the virus. It includes a seroprevalence study, which will be the second of its kind in Colombia using a probabilistic sampling design [46]. Because it will be the first to include more than one municipality, the representativeness of the sample will be increased and allow extrapolation of the results to larger populations.

It will also be the first study of population-based cohort that includes such a large sample and covers all age groups, allowing incidence to be estimated for symptomatic and asymptomatic dengue and its determinants. Asymptomatic cases have great relevance to public health, since they may act as a reservoir for dengue transmission [34]. Therefore, serological surveys are necessary to know the distribution of the transmission determinants.

Moreover, seroprevalence studies are important for the introduction of interventions such as vaccines. For example, the Strategic Advisory Group of Experts (SAGE) on immunization recommended countries introduction of the available vaccines only in settings with high endemicity, as indicated by seroprevalence of 70% or greater. On the other hand, currently, vaccine is not recommended when seroprevalence is below 50% [47].

This information together with estimates of the frequency of symptomatic underreporting of cases by the system of epidemiological surveillance, as well as, the perceived demand for vaccines by the population and circulating serotypes in the different municipalities, will assist public health officers in their decision making role [48, 49]. It will help identify vulnerable groups and modifiable factors in the human population and environment for which intervention will be useful and age groups that could benefit from vaccination, once this becomes available [42].

This study will also provide entomological information that will be help with the development and implementation of transmission interventions. The data gathered can also be nested with other entomological studies such as explore other secondary vectors, and infection with other arboviruses (eg. Chikungunya and Zika).

In that sense, ovitraps are implemented in this study as a sensitive and inexpensive method for detecting the presence of *Ae. aegypti* and *Ae. albopictus*. Monitoring vector abundance continuously using ovitraps is more sensitive than adult mosquito traps and more efficient than larval traps. In Colombia two studies have been reported using ovitramps [50, 51]. In the first, held in Cali, lethal ovitraps were used as a vector control method, but were not evaluated for their potential for the estimation of vector density or its relationship with human infection [50]. In the second evaluation was sustained for a shorter period of time (6 months) in only one municipality and a few cases of dengue were detected, which probably limited the power to identify association between the vector and dengue transmission [51].

Consequently, this will be the first study in Colombia to systematically assess ovitraps in a sustained study to assess vector density, which will be evaluated as potential predictor of transmission of infection and both febrile and asymptomatic cases. If an association is established, this tool could be used at regional and national level, facilitating surveillance and vector control.

The information gathered will improve knowledge about community attitudes which will assist in the design of educational campaigns which aim to involve the community in vector control. Early recognition of the disease will be encouraged as a strategy for appropriate management, aiming to reduce morbidity and mortality attributable to these arboviruses in the region.

While population studies have been conducted with interdisciplinary approaches to identify the determinants of dengue [52], no other projects of this magnitude have been conducted for dengue that integrates all the approaches in the same community.

We expect that this proposal will generate valuable research which will assist with the design and implementation of multidimensional strategies to reduce morbidity and mortality caused by dengue in La Guajira and other departments of the Caribbean region. We hope that knowledge about the determinants of dengue can be extrapolated to other endemic regions.

## Abbreviations

COP$: Colombian peso
DALYs: Disability-adjusted life year
DEFF: Design effect (for complex sampling)
DENV: Dengue virus
ELISA: Enzyme linked immunosorbent assay
FDC: Formats for data collection
KAP: Knowledge, attitudes and practices
US$: United States Dollar
